# Efficacy of Ibandronate Loading Dose on Rapid Pain Relief in Patients With Non-Small Cell Lung Cancer and Cancer Induced Bone Pain: The NVALT-9 Trial

**DOI:** 10.3389/fonc.2020.00890

**Published:** 2020-06-24

**Authors:** Anita J. W. M. Brouns, Lizza E. L. Hendriks, Vincent van der Noort, Ben E. E. M van de Borne, Franz M. N. H. Schramel, Harry J. M. Groen, Bonne Biesma, Hans J. M. Smit, Anne-Marie C. Dingemans

**Affiliations:** ^1^Department of Pulmonary Diseases, Zuyderland Medical Center, Sittard-Geleen, Netherlands; ^2^Department of Pulmonary Diseases, GROW—School for Oncology and Developmental Biology, Maastricht University Medical Center+ (MUMC+), Maastricht, Netherlands; ^3^Department of Biometrics, Netherlands Cancer Institute, Amsterdam, Netherlands; ^4^Department of Pulmonary Diseases, Catharina Hospital, Eindhoven, Netherlands; ^5^Department of Pulmonary Diseases, St. Antonius Hospital, Utrecht, Netherlands; ^6^Department of Pulmonary Diseases, University Medical Center Groningen, Groningen, Netherlands; ^7^Department of Pulmonary Diseases, Jeroen Bosch Hospital, ‘s-Hertogenbosch, Netherlands; ^8^Department of Pulmonary Diseases, Rijnstate Hospital, Arnhem, Netherlands; ^9^Department of Pulmonary Diseases, Erasmus MC, Rotterdam, Netherlands

**Keywords:** carcinoma, non-small-cell lung, pain management, cancer induced bone pain, clinical trials, phase II as topic, ibandronic acid

## Abstract

**Introduction:** Approximately 80% of non-small cell lung cancer (NSCLC) patients with bone metastases have cancer induced bone pain (CIBP).

**Methods:** The NVALT-9 was an open-label, single arm, phase II, multicenter study. Main inclusion criterion: bone metastasized NSCLC patients with uncontrolled CIBP [brief pain inventory [BPI] ≥ 5 over last 7 days]. Patients were treated with six milligram ibandronate intravenously (day 1–3) once a day. Main exclusion criteria: active secondary malignancy, systemic anti-tumor treatment and radiotherapy ≤4 weeks before study start, previous bisphosphonate treatment. Statistics: Simon's Optimal two-stage design with a 90% power to declare the treatment active if the pain response rate is ≥ 80% and 95% confidence to declare the treatment inactive if the pain response rate is ≤ 60%. If pain response is observed in ≤ 12 of the first 19 patients further enrollment will be stopped. Primary endpoint: bone pain response, defined as 25% decrease in worst pain score (PSc) over a 3-day period (day 5–7) compared to baseline PSc with maximum of 25% increase in mean analgesic consumption during the same period. Secondary endpoints: BPI score, quality of life, toxicity and World Health Organization Performance Score.

**Results:** Of the 19 enrolled patients in the first stage, 18 were evaluable for response. All completed ibandronate treatment according to protocol. In 4 (22.2%), a bone pain response was observed. According to the stopping rule, further enrollment was halted.

**Discussion:** Ibandronate loading doses lead to insufficient pain relief in NSCLC patients with CIBP.

## Introduction

Cancer induced bone pain (CIBP) is an important issue in metastasized non-small cell lung cancer (NSCLC). During the course of the disease, 24–60% of NSCLC patients are diagnosed with bone metastases and up to 80% will experience CIBP (1–3). Furthermore, bone metastases have a negative influence on quality of life (QoL) and are associated with a poorer overall survival (OS) (4). Radiotherapy is an effective treatment for CIBP with a 50% chance of complete pain resolution, but it unfortunately has several drawbacks. Examples are a time delay before the maximum treatment effect is obtained, the possibility of a pain flare-up, and it is only feasible in patients with a limited number of bone metastases (5). In general, pain management, according to the World Health Organization (WHO) pain ladder (6), frequently results in treatment with opioids. Especially in this vulnerable population, opioid use can result in neurologic, renal, hepatic, and/or gastro-intestinal toxicity (7).

In current guidelines [e.g., European Society for Medical Oncology [ESMO], National Comprehensive Cancer Network [NCCN], and National Institute for Health and Care Excellence [NICE]] bone targeted agents such as bisphosphonates are mentioned as an option to prevent skeletal related events (SREs) in NSCLC patients (5, 8–10). However, actual data on (rapid) pain relief of bisphosphonates are scarce in NSCLC (11). Trials including patients with bone metastases from prostate- or breast- or lung cancer (*N* = 607 of which only 1 NSCLC patient), which evaluated the effect of ibandronate (intravenous or oral) on bone pain showed pain relief within 7 days to 12 weeks after start of ibandronate (12–14). Therefore, we performed a multicenter phase II study to evaluate the effect of intravenous loading doses of ibandronate on acute pain response in NSCLC patients with uncontrolled CIBP.

## Materials and Methods

The primary aim of this open label single arm phase II study (NVALT-9, EudraCT number 2007-000885-20, NTR1602) was to establish the efficacy of intravenous loading doses of ibandronate to achieve acute bone pain relief in NSCLC patients with CIBP. The trial was approved by the appropriate ethics committee (METC 07-2-035.6/ivb).

The trial was performed in eight Dutch hospitals (see Supplementary Data, paragraph 1). The main inclusion criteria were: (I) pathologically proven NSCLC with pathologically and/or radiologically confirmed bone metastases with a patient life expectancy of at least 1 month; (II) the pain scored for bone metastases had to correspond to known locations of bone metastases (based on imaging); (III) mean bone pain score ≥ five over the last 7 days before inclusion on the worst pain scale on the brief pain inventory (BPI), (IV) use of non-steroidal anti-inflammatory drugs (NSAIDs) or a weak opioid base on the WHO analgesic ladder step 2, (V) adequate renal function (creatinine clearance as calculated by Cockcroft-Gault method > 50 ml/min). The main exclusion criteria were: (I) active secondary malignancies, (II) start of anti-tumor treatment within 4 weeks before study entry, (III) bone radiotherapy in the preceding 4 weeks, (IV) bisphosphonate treatment in the previous 2 months, (V) hypocalcemia (serum albumin corrected calcium concentration <2 mmol/L) or hypercalcemia (serum albumin corrected calcium ≥ 2.7 mmol/L).

With the aim of assessing the efficacy of ibandronate on acute bone pain relief, the primary endpoint was acute bone pain response over a 7-day period. This was defined as a 25% decrease in worst bone pain score over day 5, 6, and 7 compared to bone pain score at baseline (determined by the “worst pain scale” of the BPI), with no more than a 25% increase in mean analgesic consumption over the same 3-day period compared to baseline analgesic consumption. Secondary endpoints were mean worst bone pain scale of the BPI in the first 7 days, interference scales of the BPI, analgesic consumption, WHO-Performance Score (WHO-PS), QoL, and safety. In the context of safety spontaneous adverse events (scored by Common Terminology Criteria for Adverse Events [CTCAE] version 4.0) were collected.

Patients were treated with six mg ibandronate once a day intravenously on day one, two, and three. Concomitant analgesic use was assessed using the WHO pain ladder. On day 1–7 patients recorded their worst bone pain score of the BPI and their analgesic consumption in a diary. Patients were evaluated for BPI, WHO-PS, and QoL on day 1 and 7. On day 7, a serum chemistry panel (including serum creatinine) was performed to assess the renal safety of ibandronate. Adverse events were recorded on day 1–3, on day 7, and at the end of follow-up (day 28).

The study was designed following a Simon's Optimal two-stage design with a 90% power to declare the treatment active if the pain response rate was ≥ 80 and 95% confidence to declare the treatment inactive if the pain response rate was ≤ 60%. The 60% pain response rate is comparable to expected response rates of radiotherapy and opiates on cancer related pain (5, 15). In the first stage, 19 patients were treated and evaluated. If ≤ 12 pain responses were observed the study was stopped. Otherwise, 34 patients would subsequently be enrolled and the treatment would be declared active if ≥38 of 53 included patients had a positive pain response.

## Results

**Figure 1:**
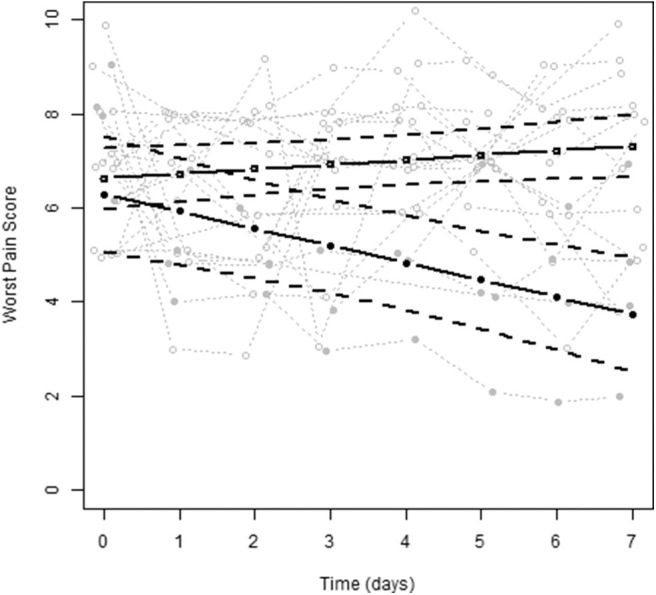
The observed and estimated worst pain scores for patients grouped by outcome (no bone pain responder vs. bone pain responder). No bone pain response is depicted by open circles (*N* = 14), bone pain response is depicted by solid circles (*N* = 4).

Between December 2007 and November 2010, 19 NSCLC patients were enrolled in the first stage. Eighteen out of 19 patients were evaluable for response. The patient characteristics are shown in Table 1. One patient received only 1 day of study medication, as it was discovered that the patient was ineligible because of previous bisphosphonate use. All other patients received all doses of ibandronate without dose reductions or dose delays. Patients were treated with analgesics according the WHO pain ladder (6). Before study entry, four patients were also treated with anti-epileptics and one patient with methadone because of uncontrolled pain. Except for two patients, none of the patients were able to reduce their analgesic use during the study period and in two other patients, the opioid doses increased. For the primary endpoint of bone pain response, four out of 18 patients (22%) had a ≥25% decrease in worst pain score over day 5–7, therefore the endpoint was not met and the study was discontinued. Figure 1 shows the observed and estimated worst bone pain scores for patients grouped by outcome.

**Table 1:** Patient characteristics.

**Patient characteristics**		***N =* 18**
Gender, male (%)		12 (67)
Age, mean (range)		58.7 (42–74)
WHO-PS at baseline (%)	0	1 (6)
	1	11 (61)
	2	2 (11)
	3	2 (11)
	Unknown	2 (11)
Prior treatment for malignancy	Surgery for BM (%)	2 (11)
	Chemotherapy (%)	12 (67)
	Palliative radiotherapy to BM, yes (%)	5 (28)
Current analgesics (n/N)	Analgesics according to WHO pain ladder^a^	15/15
	Analgesics according to WHO pain ladder^a^ + anti-epileptics	4/15
	Analgesics according to WHO pain ladder^a^ + anti-epileptics + methadone	1/15
Patient status at day 28 (%)	Alive	9 (64)
Patient status at day 28 for bone pain responders (%)	Alive	4 (100)

*WHO-PS, World Health Organization Performance Score; BM, bone metastases*.

a *Analgesics according to WHO pain ladder means a one to three step, which starts with non-opioids with or without any adjuvant therapy and increases to opioids for moderate to severe pain with or without any non-opioids or adjuvant therapy*.

In Table 2 primary and secondary endpoints were shown. For secondary endpoints, two patients (11.1%) reported an improvement of WHO-PS during treatment while 33.3 and 16.7% reported no change or a worsening of WHO-PS, respectively. In seven patients (38.9%) change in WHO-PS was not recorded. No relation between change in WHO-PS and bone pain response was recorded. After 28 days follow-up 5 patients (27.8%) had died. Mean worst bone pain scale of the BPI, interference scales of the BPI, analgesic consumption, and QoL revealed no significant or clinically relevant differences between study participants (see Supplementary Data, paragraph 2). No serious adverse events were reported. Two patients experienced ≥ grade 2 hypophosphatemia (one grade 2, one grade 3), which was possibly treatment related.

**Table 2:** Primary and secondary endpoints.

**Primary endpoint**		
Bone pain response^a^ N, %	Yes	4 (22)
**Secondary endpoint**		
Mean worst pain score over last seven days at baseline^b^ (%)	5 6 7 8 9 10	4 (22) 2 (11) 5 (28) 4 (22) 2 (11) 1 (96)
Interference scales of the BPI	No significant or clinically relevant difference between scores at day 1 compared to day 7 on all interference scales of the BPI.	
Analgesic consumption	No significant or clinically relevant difference between analgesic consumption at day 1 compared to day 7	
Change in WHO-Performance Score day 1 compared to day 6N, %	No change Improvement Worsening Not reported	6 (33) 2 (11) 3 (17) 7 (39)
Quality of Life^c^	No significant or clinically relevant difference between scores at day 1 compared to day 7 on all dimensions of QLQ-C30 questionnaire.	
Spontaneous adverse events N, %	CTCAE grade 2 hypophosphatemia	1 (6)

*BPI, brief pain inventory; QLQ-C30, Quality of Life Questionnaire-Core 30; CTCAE v4.0, Common Terminology Criteria for Adverse Events*.

a *definition of bone pain response: a 25% decrease in worst bone pain score over day five, six, and seven compared to bone pain score at baseline (as determined by the “worst pain scale” of the BPI), with no more than a 25% increase in mean analgesic consumption over the same three-day period compared to baseline analgesic consumption*.

b *measured on a scale 0–10 according to the brief pain inventory*.

c *scored by Quality of Life Questionnaire-Core 30 (QLQ-C30)*.

## Discussion

This study of intravenous loading doses of ibandronate in NSCLC patients with CIBP did not show adequate pain reduction in most patients. However, in four out of 18 patients a sufficient pain response was observed. In contrast to most other trials in which patients used concomitant anti-cancer therapy (making evaluation of the effect of the bone targeted agent more difficult), patients in this study were only eligible if they did not start an anti-cancer therapy (systemic therapy and/or radiotherapy) in the 4 weeks before study entry. To not unnecessarily expose patients to a treatment that was not beneficial enough, a Simon 2-stage design was used, and the trial was stopped due to futility.

CIBP remains important in NSCLC. Despite these older data of ibandronate loading dose for acute pain relief in lung cancer patients with CIBP, no new treatment options with rapid pain relief are currently available. Furthermore, phase II/III trials in metastasized NSCLC evaluating systemic anti-cancer treatment modalities (i.e., chemotherapy with or without immunotherapy and tyrosine kinase inhibitors) are not focused on rapid bone pain reduction and only report pain as an adverse event of therapy. We show that loading doses of bisphosphonates do not induce a rapid reduction of CIBP and that other strategies should be pursued.

There is evidence of the effects of bisphosphonates on pain reduction for breast cancer, multiple myeloma and prostate cancer (12–14, 16). Analogous to a previous pilot study (13), in which opioid refractory bone pain was relieved by ibandronate loading doses within 7 days, ibandronate was the bisphosphonate of choice in our study. It is unclear why breast or prostate cancer patients responded to loading doses of bisphosphonates in a previous studies (12–14, 17, 18), while the NSCLC patients in our study did not. There could be different explanations why NSCLC patients in this study do not respond to bisphosphonates: (I) Influence of tumor histology on the chance of bone pain reduction by bisphosphonates, (II) Possible differences in the metabolism of bone metastases between breast cancer and lung cancer (although not shown when evaluating bone turnover markers) (19) or (III) Lack of concomitant systemic therapy which resulted in reduced pain control, as in a systematic review and meta-analysis the combination of bisphosphonates and systemic therapy was superior compared with one of the two treatment modalities alone (20).

We observed that bone pain response was not associated with an improvement in WHO**-**PS; only one of the bone pain responding patients improved while two patients with a reduction in pain had a deteriorating WHO-PS, probably due to progression of cancer. Bone pain response was associated with survival as all four patients in the bone pain responding group were alive at study completion, whereas five of the 14 patients in the no bone pain responding group already had died because of progressive disease. Of all enrolled patients, 27.8% died within 1 month, although only patients with a life expectancy of more than 1 month could be enrolled. This stresses that it is difficult for physicians to accurately estimate the prognosis of a patient. It is already known that physicians tend to overestimate survival of patients in 27–42% of patients (21, 22). However, 2/3 of the included patients in our study had a good performance status (WHO PS 0-1), and in general this is associated with a survival of more than 1 month (23). Compared with the literature, QoL was lower for our patients, especially on the domains of role, cognitive, and social functioning (24–26). It could be that the high pain scores influenced these parts of QoL.

## Limitations and Strengths

A limitation of this study is the BPI as assessment tool of bone pain, because the BPI does not exclude pain from other causes. To the best of our knowledge, there are no tools or questionnaires to fully discriminate between bone pain and pain from other causes. We attempted to minimize bias due to pain from other causes by only including patients with bone metastases diagnosed by imaging studies, bone pain corresponding with a location of bone metastases on imaging, or investigator judgment that the reported pain was indeed caused by bone metastases. Lacking a placebo arm is another limitation of the study. However, in light of insufficient pain relief by ibandronate, this probably did not have any influence on the interpretation of the results (i.e., low chance of placebo effect). Furthermore, we assessed changes in WHO-PS at 7 days after ibandronate infusion. Additional collection of WHO-PS through day 28 would have likely added value in identifying long-term changes in performance status. A strength of this study is the separation of systemic treatment and treatment for CIBP as there is no potential interaction in efficacy on rapid pain relief.

## Conclusion

In conclusion, loading doses of ibandronate do not lead to rapid bone pain relief in a sufficient number of NSCLC patients with uncontrolled CIBP to constitute its use. Studies evaluating other treatment options for rapid bone pain relief in this patient population are necessary.

## Data Availability Statement

The datasets generated for this study are available on request to the corresponding author.

## Ethics Statement

The studies involving human participants were reviewed and approved by Medisch ethische toetsings commissie (METC) Maastricht. METC 07-2-035.6/ivb. The patients/participants provided their written informed consent to participate in this study.

## Author Contributions

A-MD and BEEMB: conceptualization. VN: formal analysis. AB: writing-original draft preparation. LH, VN, BEEMB, FS, HG, BB, and HS: writing-review & editing. All authors agree to be accountable for the content of the work.

## Conflict of Interest

A-MD has taken part on an advisory board for Roche. The remaining authors declare that the research was conducted in the absence of any commercial or financial relationships that could be construed as a potential conflict of interest.
