# Supplementary material for: Efficacy of Ibandronate Loading Dose on Rapid Pain Relief in Patients With Non-Small Cell Lung Cancer and Cancer Induced Bone Pain: The NVALT-9 Trial
Source: Front Oncol. 2020 Jun 24;10:890. doi: 10.3389/fonc.2020.00890 (PMC7326766; doi:10.3389/fonc.2020.00890)
Supplement: Supplementary file 1 [file Data_Sheet_1.PDF]

Supplementary Material

1 Participating centers of the NVALT-9 trial.

Participating centers of the NVALT-9 trial are:

1. Maastricht University Medical Center+ (MUMC+), Maastricht, The Netherlands.
2. Catharina Ziekenhuis, Eindhoven, The Netherlands.
3. Sint Antonius Ziekenhuis, Nieuwgein, The Netherlands.
4. Viecuri, Venlo, The Netherlands.
5. Rode Kruis Ziekenhuis, Beverwijk, The Netherlands.
6. Jeroen Bosch Ziekenhuis, ’s Hertogenbosch, The Netherlands.
7. Leids Universitair Medisch Centrum, Leiden, The Netherlands.
8. Rijnstate Ziekenhuis, Arnhem, The Netherlands.

2 Analysis of secondary endpoints.

2.1 Mean worst bone pain scale of the BPI.

Table 1: mean worst bone pain scale of the BPI.

	BPI day 1<br>n/N=18/18	BPI day 7<br>n/N=16/18
Worst pain<br>Median (range)	7.5 (4.0-9.0)	7.0 (2.0-9.0)

Abbreviation: BPI: brief pain inventory.

To conclude: no significant or clinically relevant difference.

2.2 Interference scales of the BPI.

Table 2: interference scales of the BPI.

	BPI day 1<br>n/N=18/18	BPI day 7<br>n/N=16/18
Average pain<br>Median (range)	5.0 (2.0-9.0)	5 (2.0-7.0)
General activity<br>Median (range)	6.0 (3.0-10.0)	8.0 (2.0-9.0)
Mood<br>Median (range)	5.0 (1.0-10.0)	6.0 (0.0-8.0)
Ability to walk<br>Median (range)	7.0 (1.0-10.0)	8.0 (1.0-10.0)

Normal operation<br>Median (range)	8.0 (2.0-10.0)	8.5 (3.0-10.0)
Relationships with others<br>Median (range)	5.0 (0.0-10.0)	4.0 (0.0-9.0)
Sleep<br>Median (range)	7.0 (0.0-10.0)	4.0 (0.0-9.0)
Pleasure in life<br>Median (range)	5.0 (1.0-10.0)	5.0 (1.0-8.0)

Abbreviation: BPI: brief pain inventory.

To conclude: no significant or clinically relevant difference.

2.3 Analgesic consumption.

Patients were treated with analgesics according the WHO pain ladder. Before study entry, four patients were also treated with anti-epileptics (4 patients) and methadone (1 patient).

Two patients were able to reduce their analgesic use during the study period (one stopped opioid treatment, the other the dose decreased with 25%). In two other patients the opioid doses increased substantially (times 2 in one patient, times 5 in the other patient).

To conclude: no significant or clinically relevant difference.

2.4 WHO-PS.

Table 3: WHO-PS change in general.

		n/N=18/18 (%)
WHO-PS change	No change	6 (33)
	Improvement	2 (11)
	Worsening	3 (17)
	Not reported	7 (39)

Abbreviation: WHO-PS: World Health Organization Performance Score.

Table 4: WHO-PS change subdivided in no bone pain responder vs. bone pain responder*.

		No bone pain responder n/N=14/18 (%)	Bone pain responder n/N=4/18 (%)
WHO-PS change	No change	6 (33)	0 (0)
	Improvement	2 (11)	1 (25)
	Worsening	3 (17)	2 (50)
	Not reported	7 (39)	1 (25)

Abbreviations: WHO-PS: World Health Organization Performance Score.
*Definition of a bone pain responder: a 25% decrease in worst bone pain score over day five, six and

seven compared to bone pain score at baseline (as determined by the "worst pain scale" of the BPI), with no more than a 25% increase in mean analgesic consumption over the same three-day period compared to baseline analgesic consumption.

To conclude: no significant or clinically relevant difference.

2.5 Quality of life

Table 5: QLQ-C30 scores in general.

	QLQ-C30 day 1<br>n/N=18/18	QLQ-C30 day 7<br>n/N=16/18
Global QoL<br>Median (range)	33.3 (0.0-66.7)	33.3 (16.7-66.7)
Physical functioning dimension<br>Median (range)	40.0 (13.3-86.7)	33.3 (6.7-80.0)
Role functioning dimension<br>Median (range)	16.7 (0.0-50.0)	0.0 (0.0-66.7)
Emotional function dimension<br>Median (range)	50.0 (0.0-91.7)	58.3 (8.3-83.3)
Cognitive functioning dimension<br>Median (range)	66.7 (0.0-100.0)	66.7 (0.0-100.0)
Social functioning dimension<br>Median (range)	41.7 (0.0-100.0)	33.3 (0.0-100.0)

Abbreviations: QLQ-C30: Quality of Life Questionnaire-Core 30; QoL: Quality Of Life.

To conclude: no significant or clinically relevant difference.

Table 6: QLQ-C30 scores subdivided in QLQ-C30 symptom scores of greater than 50% maximum attainable value.

	QLQ-C30 day 1<br>n/N=18/18 (%)	QLQ-C30 day 7<br>n/N=16/18 (%)
Nausea > 50%	1 (5)	1 (5)
Pain > 50%	14 (74)	11 (58)
Dyspnea > 50%	6 (32)	6 (32)
Insomnia > 50%	10 (53)	6 (32)
Appetite loss >50%	7 (37)	7 (37)
Constipation >50%	6 (32)	6 (32)
Diarrhea >50%	1 (5)	3 (16)
Financial difficulties > 50%	4 (21)	3 (16)

Abbreviations: QLQ-C30: Quality of Life Questionnaire-Core 30.

To conclude: no significant or clinically relevant difference.
